# Proteomic Characterization of Two Extracellular Vesicle Subtypes Isolated from Human Glioblastoma Stem Cell Secretome by Sequential Centrifugal Ultrafiltration

**DOI:** 10.3390/biomedicines9020146

**Published:** 2021-02-03

**Authors:** Fabrizio Di Giuseppe, Marzia Carluccio, Mariachiara Zuccarini, Patricia Giuliani, Lucia Ricci-Vitiani, Roberto Pallini, Paolo De Sanctis, Roberta Di Pietro, Renata Ciccarelli, Stefania Angelucci

**Affiliations:** 1 Department of Innovative Technologies in Medicine and Dentistry, ‘G. d’Annunzio’ University of Chieti-Pescara, Via Vestini 31, 66100 Chieti, Italy; f.digiuseppe@unich.it; 2 Center for Advanced Studies and Technology (CAST), ‘G. d’Annunzio’ University of Chieti-Pescara, Via L Polacchi 13, 66100 Chieti, Italy; marzia.carluccio@unich.it (M.C.); mariachiara.zuccarini@unich.it (M.Z.); patricia.giuliani@unich.it (P.G.); paolo.desanctis@studenti.unich.it (P.D.S.); roberta.dipietro@unich.it (R.D.P.); renata.ciccarelli@unich.it (R.C.); 3 Stem TeCh Group, Via L Polacchi 13, 66100 Chieti, Italy; 4 Department of Medical, Oral and Biotechnological Sciences, ‘G. d’Annunzio’ University of Chieti-Pescara, Via Vestini 31, 66100 Chieti, Italy; 5 Department of Oncology and Molecular Medicine, Istituto Superiore di Sanità, Via Regina Elena 299, 00161 Rome, Italy; lucia.riccivitiani@iss.it; 6 Institute of Neurosurgery, Università Cattolica del Sacro Cuore, Largo Agostino Gemelli 8, 00168 Rome, Italy; roberto.pallini@unicatt.it; 7 Department of Medicine and Ageing Sciences, ‘G. d’Annunzio’ University of Chieti-Pescara, Via Vestini 31, 66100 Chieti, Italy

**Keywords:** glioblastoma (GBM), glioblastoma-derived stem-like cells (GSCs), sequential centrifugal ultrafiltration (SCUF), microvesicles, oncosomes, exosomes, proteomics

## Abstract

Extracellular vesicles (EVs) released from tumor cells are actively investigated, since molecules therein contained and likely transferred to neighboring cells, supplying them with oncogenic information/functions, may represent cancer biomarkers and/or druggable targets. Here, we characterized by a proteomic point of view two EV subtypes isolated by sequential centrifugal ultrafiltration technique from culture medium of glioblastoma (GBM)-derived stem-like cells (GSCs) obtained from surgical specimens of human GBM, the most aggressive and lethal primary brain tumor. Electron microscopy and western blot analysis distinguished them into microvesicles (MVs) and exosomes (Exos). Two-dimensional electrophoresis followed by MALDI TOF analysis allowed us to identify, besides a common pool, sets of proteins specific for each EV subtypes with peculiar differences in their molecular/biological functions. Such a diversity was confirmed by identification of some top proteins selected in MVs and Exos. They were mainly chaperone or metabolic enzymes in MVs, whereas, in Exos, molecules are involved in cell–matrix adhesion, cell migration/aggressiveness, and chemotherapy resistance. These proteins, identified by EVs from primary GSCs and not GBM cell lines, could be regarded as new possible prognostic markers/druggable targets of the human tumor, although data need to be confirmed in EVs isolated from a greater GSC number.

## 1. Introduction

For many decades, it has been thought that communications among cells relied on soluble molecules released from the cells themselves, which can act in autocrine/paracrine/endocrine fashions. However, it is now evident that another important route assures an intense intercellular exchange of information via extracellular vesicles (EVs), which comprise endosome-derived exosomes (Exos, 30–100 nm size) and plasma membrane-derived micro-vesicles (MVs, 100–1000 nm size). These nanoparticles are secreted by virtually all cell types and carry a heterogeneous number of substances, deriving from intracellular compartments and including different types of nucleic acids, proteins, and lipids. In this way, circulating EVs reflect the identity and the molecular state of their cell-of-origin [1].

In the last decade, a plethora of information about EV composition and molecular function has emerged, along with the notion that cancer cells also release these particles, relying on them to invade tissues and propagate oncogenic signals at distance. Exos and MVs may contribute to the distribution of cancers, also modifying the cells in the tumor niche and leading them to transformation [2]. Of interest, in tumors, besides apoptotic bodies (1000–5000 nm) released from cells undergoing programmed cell death [3], a novel population of large EVs, named oncosomes, has been identified. These vesicles have a diameter of 1–10 µm and seem to contain oncogenic material and to be cancer-specific [4,5].

The knowledge on biogenesis, molecular content, and horizontal communication of EVs in diverse types of cancer, including glioblastoma (GBM), has expanded considerably in recent years [6]. GBM is the most common and lethal neuroepithelial primary brain tumor in humans, belonging to the family of gliomas [7]. GBM is characterized by high cell proliferation rate and infiltrating capacity of the surrounding tissues, against which the current therapy (i.e., surgical removal combined with radio- and chemo-therapy) is largely ineffective, thus contributing to its worse prognosis [8]. These features seem to be closely related to the presence of stem-like cells inside the tumor mass, which are therefore called GBM stem-like cells (GSCs). Once isolated from primary GBM surgical samples and cultured in vitro, these cells show stem cell marker expression, high self-renewal, and resistance to radiation/chemotherapy agents [9,10], thus reproducing phenotype/genotype characteristics of the primary tumor better than GBM cell lines [11,12]. Additionally, if orthotopically injected into mice brains, GSCs give rise to tumors recapitulating features of the human ones [13]. Therefore, they are considered a proper model to investigate GBM new peculiarities, potentially useful as new therapeutic targets.

It is now clear that GBMs are able to release EVs [14], thanks to studies that have identified many of the intracellular molecules secreted by EVs. Investigation has been mostly performed on EVs deriving from different human glioma cell lines [15,16] and, also, in GSCs [17,18,19] or plasma from patients with primary GBM [20]. However, it has been limited to isolation and characterization of Exos. Since GBMs may secrete different EV types as mentioned above, we thought that it would be important to evaluate the content of these other particles in order to implement knowledge about molecules therein contained, which, in turn, could allow a stratification useful for GBM prognosis assessment as well as for monitoring of treatment response.

Based on these premises, we started a new investigation aimed at identifying and characterizing by a morphological and biological point of view the types of EVs released from GSCs in vitro. In particular, for the first time, we performed a proteomic comparison between two different EV subpopulations isolated from the conditioned medium (CM) of GSCs, in order to get a more global protein profile of the secretome from these cells. Hopefully, the identified tumor-related proteins in our study could be used as reference to develop targeted strategies for a better diagnosis and clinical treatment of GBM.

## 2. Materials and Methods

### 2.1. Materials and Chemicals

Disposables materials for tissue culture were from Falcon (Steroglass, Perugia, Italy). Dulbecco’s Modified Eagle’s Medium/Nutrient Mixture F-12 Ham (DMEM/F-12) was purchased from Sigma-Aldrich S.p.A. (Milan, Italy) as well as penicillin/streptomycin, amphotericin B, and all the other chemicals, unless differently indicated. Human epidermal (EGF) and fibroblast (FGF) growth factors were purchased from PeproTech (SIAL, Rome, Italy). Protease inhibitors Mix, Immobiline Dry Strip 4–7 IPG (acrylamide gel), the Dry Strip cover fluid (98% (*v*/*v*) liquid paraffin solution), De STREAK Reydratation Solution (IEF running buffer), buffer IPG 4–7 (40% ampholin), and agarose were purchased from GE Healthcare (Uppsala, Sweden). 2.7 Disulfonicnaphthalenic acid (NDS) Acros, and porcine trypsin was provided by Thermo Fisher Scientific Geel, Belgium, and Promega Bioscience, CA, respectively. All the solutions used were prepared with Milly-Q water (Millipore, Bedford, MA, USA).

### 2.2. Cell Cultures

The experiments were carried out following the rules of the Declaration of Helsinki on GSCs, the same used in previous studies [21,22]. They were obtained from two different patients with primary GBM [23,24], who provided a written informed consent to the study according to research proposals approved by the institutional Ethics Committee of the Catholic “Sacro Cuore” University (UCSC) School of Medicine (Prot. 4720/17 approved on March 16, 2017). In this paper, we indicated as GSCs # 1 those deriving from the patient # 1, whereas GSCs # 2 correspond to cells from the patient # 83.

These cells have previously been characterized for some crucial features such as in vitro self-renewal potential, constant expression of stemness markers, and resistance to chemotherapy drugs [23,24]. Moreover, when injected in immune-compromised mouse brain, they reproduced a tumor identical to the human one as for antigen expression and histological tissue organization [25].

Upon their isolation from the GBM tissue, cells were grown in serum-free medium, supplemented with mitogens (20 ng/mL of human recombinant EGF and 10 ng/mL of human recombinant FGF-basic), as previously described [26]. Under these conditions, cells formed classical floating neurospheres, which were used to expand them in vitro. For our experiments, we seeded a very great number of GSCs (2 × 10^9^ cell) on culture flasks (175 mL) pre-coated with Matrigel (Corning, SIAL) that had been dissolved in culture medium. In this condition, cells grew as a monolayer that allowed a more precise quantification of in vitro survival of GSCs, leaving their spherogenic properties unaltered [27]. Cells were fed with the usual culture medium above described for 48 h; subsequently, they were cultured for further 48 h in DMEM/F-12 Ham medium containing low glucose concentration (1000 mg/L) and no phenol red (Sigma-Aldrich), and supplemented with the same mitogens above cited. This CM was removed and used for EV isolation and characterization. GSCs were used from passage 5 to 10 throughout the study. No significant modification in cell morphology was found in these cultures over the indicated passages.

### 2.3. EV Isolation by Sequential Centrifugal Ultra-Filtration

**Figure 1:**
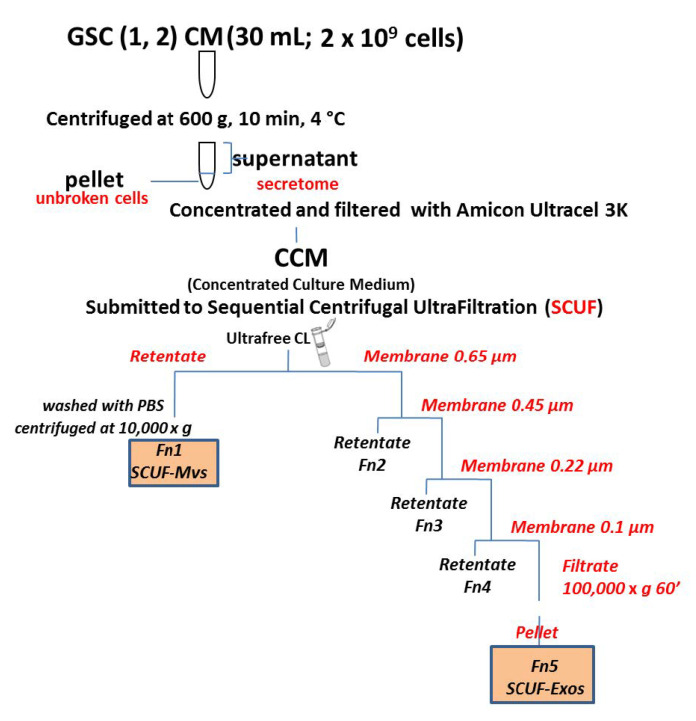
Isolation procedures of EVs from the conditioned medium (CM) of GSCs. Flow chart of the experimental steps followed to isolate two different subtypes of EVs from the CM of glioblastoma stem-like cells (GSCs) using the sequential centrifugal ultrafiltration (SCUF) method.

The EV components were isolated from the CM of GSCs by SCUF, according to the procedure described by Xu R et al. [28], and partially modified as shown in the work flow (Figure 1). Briefly, the CM (30 mL; 2 × 10^9^ cells) was concentrated to 2 mL (concentrated culture medium, CCM) and filtered with Amicon Ultracel3K (Millipore, Merck KGaA, Darmstadt, Germany). Subsequently, the vesicular fractions were separated by the following steps, using different pore-sized ultrafilters (Durapore Ultrafree CL, Merck Millipore) from 0.65 to 0.45, 0, 22, and 0.1 µm:

1. centrifugation of 2 mL of CCM at 3000× *g* combined with the use of 0.65 μm ultrafilters;
2. transfer of the non-filtered fraction into a microtube with 0.5 mL of PBS and centrifugation at 10,000× *g* for 30 min. The particles isolated from this step were then characterized as MVs or Fn1 fraction;
3. sequential filtration of the fraction obtained from the passage 1 through 0.45, 0.22, and 0.1 μm filters;
4. ultrafiltration of the last filtered fraction at 100,000× *g* for 1 h. The particles isolated from this step were then characterized as Exos or Fn5 fraction;
5. re-suspension of all preparations with 500 μL of phosphate buffered saline (PBS) and subsequent protein lysis of each fraction to perform two-dimensional electrophoretic analysis.

The protein concentration of each sample was determined using the BCA Pierce method assay [29].

### 2.4. Electron Microscopy of Isolated EVs

Samples were processed by TEM, as previously reported [30,31]. Briefly, pellets obtained by SCUF (Fn1 and Fn5) were carefully fixed with 2.5% glutaraldehyde in 0.1 M cacodylate buffer (pH 7) for 1 h at 4 °C. Samples were post-fixed with 1% osmium tetroxide (Electron Microscopy Sciences, Fort Washington, WA, USA) for 1 h at 4 °C, dehydrated with a graded acetone series (from 50% to 100%), and embedded in Spurr resin. Semithin sections were cut with a Powertome X RMC ultramicrotome (Science Services, Fort Washington, WA, USA), stained with 1% toluidine blue solution, and analyzed under a ZEISS Axioskop 40 (Carl Zeiss, Göttingen, Germany) light microscope, equipped with Coolsnap Videocamera (Photometrics, Tucson, AZ, USA). Ultrathin sections were stained with uranyless (Electron Microscopy Sciences, Fort Washington, WA, USA) and lead citrate. Samples were observed under a Zeiss EM109 electron microscope, and ultrastructural images were acquired with a GATAN Fastcam 830 CCD camera (Gatan Inc., Pleasanton, CA, USA).

### 2.5. Western Blot Analysis

This method was applied to the isolated vesicular fractions (Fn1 and Fn5) and the cell pellet to reveal markers possibly specific for each of them and to exclude, where possible, contamination by proteins deriving from cell debris.

Following a classic procedure, protein samples (30 μg) were diluted in sodium dodecylsulphate (SDS)-bromophenol blue buffer, boiled (5 min), and separated on 10% SDS polyacrylamide gel (PAGE). Resolved proteins were transferred on polyvinylidene fluoride membrane, then blocked with PBS/0.1% Tween 20/5% nonfat milk (Bio-Rad Laboratories, Hercules, CA, USA) for 2 h at 4 °C and overnight incubated at 4 °C with primary antibodies (polyclonal rabbit anti-ALIX, catalog n. SAB4200476, dilution 4 mg/mL; polyclonal rabbit anti-Calnexin, catalog n. 208880, dilution 1:2000; polyclonal rabbit anti-CD63, catalog n. SAB2109138, dilution 1:1000 and monoclonal rabbit anti-EPCAM, catalog n. ZRB1215, dilution 1:200), all purchased from Sigma-Aldrich. Subsequently, the membranes were incubated for 1 h at room temperature to goat anti-rabbit HPR-conjugated secondary antibody (final dilution 1:5000, Bethyl Laboratories Inc.; Montgomery, TX, USA). Immunocomplexes were visualized by chemiluminescence (ECL) detection system (GE Healthcare Life Sciences, Milan, Italy) and quantified by densitometric analysis (ImageJ software; U.S. National Institutes of Health, Bethesda, MD, USA).

### 2.6. 2DE Analysis

Comparative 2DE analysis was carried out on total cell extracts from GSCs of two patients with primary GBM by 2DE and MALDI TOF MS/MS to define the microvesicular and exosomal proteomes. Each sample was electrophoretically run two times as a biological replicate. The cell pellet and the fractions Fn1 and Fn5, obtained by extraction and subsequent lysis procedures, were loaded on Immobiline Dry Strip IPG 4–7, 24 cm overnight in rehydration mode on Etthan IPGphorIEF System (Cytiva, formerly GE Healthcare, Freiburg, Germany). The amount of proteins loaded was 150 µg for the analytical gels and 500 µg for the preparative gels. The IPG strip gels were subsequently applied on homogeneous acrylamide gel, SDS PAGE (12%), in a 0.5% (*w*/*v*) agarose solution containing blue phenol bromide. The electrophoretic run was carried out at 170 W constant for 6 h, using the multiple charging system “Dodeca Cell” (Bio Rad Laboratories, Hercules, CA, USA).

Analytical gels were then stained with ammoniacal silver nitrate, while gels used for protein identification by MALDI-TOF mass spectrometry (MS) were silver-stained without glutaraldehyde, in accordance to the mass compatible method described by Angelucci et al. [32]. Subsequently, the scanned gels were digitized by LabScan 5.0 software (GE Healthcare, Uppsala, Sweden) in transparency mode at 600 dpi. The digital images of the gels were analyzed by 2D Platinum 6.0 Image Master software (GE Healthcare, Uppsala, Sweden).

The gel position calibration was performed using the 2D calibration method, calculating the position of the protein spots according to their isoelectric point (pI) and molecular weight.

**Figure 2:**
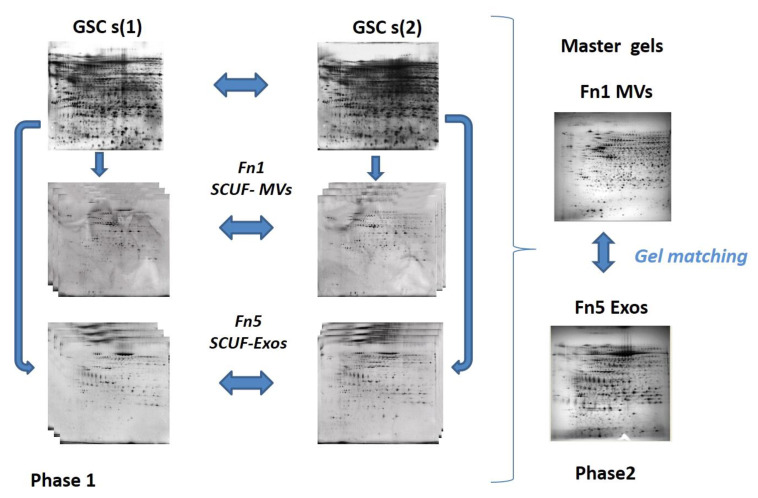
2D analysis on proteomes of the two EV types isolated from the CM of GSCs (1, 2) by two patients with primary GBM. Phase 1 includes comparative analysis between biological replicates from Fn1 and Fn5 in order to create representative gels, so called master gels that are matched in Phase 2.

In order to create a representative 2D map of both vesicular fractions analyzed, Fn1 and Fn5, gels from all technical and biological replicates were compared. The reference gel was then used to determine the expression and the difference in protein expression among all gels (Figure 2). We performed background subtraction and normalised the intensity volume of each spot with the total intensity volume (summing up the intensity volumes obtained from all spots within the same 2-D gel). All quantitative data are reported as average value ±SEM. The intensity of each spot on all gels, corresponding to the biological replicates for the conditions analyzed, was defined by comparative analysis among clusters and validated by ANOVA (statistical analysis of the variables) test. Protein spots with a statistically significant expression level (*p* < 0.001) were selected for identification by MALDI-TOF-TOF MS.

### 2.7. Protein Digestion and MALDI TOF MS/MS Analysis

The protein spots of interest were excised from the gel, analyzed by the “peptide mass fingerprinting” (PMF) method and validated by LIFT-MS/MS.

More in detail, the protein spots, once isolated from the gel, were washed with Milli-Q water for 10 min and then bleached with a solution (1:1) composed of potassium ferricyanide 30 mM and sodium thiosulphate 100 mM. After three washes in water, the spots were treated with 200 mM ammonium bicarbonate (NH_4_HCO_3_), alkylated with iodoacetamide (IAA) 55 mM, and reduced with dithiothreitol (DTT) 10 mM. Following a more intense dehydration with 100% acetonitrile (ACN), the enzymatic digestion of each spot was carried out, resuspending it in a solution of NH_4_HCO_3_ 5 mM and trypsin, first kept on ice for 30 min and then incubated at 37 °C for 12 h.

The concentration and desalination of the extracted peptides took place in a chromatographic microsystems C18ZipTip (Millipore, Bedford, MA, USA), in order to eliminate any interferent capable of invalidating the analysis of the tryptic digest thus obtained. Treatment with C18ZipTip involved repeated washing with 0.1% trifluoroacetic acid (TFA) and elution in 0.5 µL of a saturated solution of α-cyan-4hydroxycinamic acid (1:1 = HCCA: 0.1% TFA), applied directly on the ground-steel and suitable for MS investigations by the AUTOFLEX Speed MALDI-TOF/TOF MS instrument (Bruker Daltonics, Staufen, Germany), previously calibrated with external standards, such as Bradykinin (fragment 1–7) 757.39 *m*/*z*, Angiotensin II 1046.54 *m*/*z*, ACTH (fragment 18–39) 2465.19 *m*/*z*, [Glu-1]-Fibronepeptide B 1571.57 *m*/*z*, and porcine renin tetradecapeptide substrate 1760.02 *m*/*z*. The proteins picked and digested produced a spectrum in PMF analysis with a range beyond *m*/*z* 700–3000 Da. The PMF data, put into a database using the Mascot search engine, allowed us to compare masses obtained experimentally from the tryptic digest of protein selected from gels with molecular mass larger than 10,000 Dalton.

Each spectrum was obtained through the accumulation of data from 100 laser shots to obtain a range beyond *m*/*z* 700–3000 Da. The internal calibration of the mass was carried out using the trypsin autolysis products (842.50 *m*/*z*, 1.045.56 *m*/*z*, 2.211.11 *m*/*z*, 2.283.19 *m*/*z*). The peaks of trypsin and keratin contaminants were eliminated from the peak list through a research database.

The PMF result was put into a database (NCBI and Swiss Prot) through the Mascot search engine, which compares the masses obtained experimentally from the tryptic digest with the theoretical masses calculated from the database. The research parameters were the following: peptide mass finger printing, trypsin, fixed modifications such as carbamido-methylation (Cys), variable changes such as oxidation of methionine, monoisotopic mass, state of charge of the peptide +1, maximum number of errors in peptide cutting up to 1, mass tolerance for each peptide at 100 ppm and 0.6 and 0.8 daltons for MS/MS. Subsequently, protein assignment was validated using LIFT-MS/MS technology, selecting the most abundant ones as ions to be subjected to MS/MS analysis.

A maximum number of precursor ions per sample equal to 4 were chosen. The database search through Mascot was based on the use of combined PMF and MS/MS data using the BioTools 3.2 program connected to the Mascot search engine. The probability score that corresponds to a match between the experimental data and each sequence deposited in the database with *p* < 0.05 was used as a criterion for correct identification. The scores were reported as log10 (P), where P represents the maximum probability. The acceptable score value was set at 70 for PMF and 30/40 for MS/MS research.

### 2.8. Bioinformatic Analysis

To understand the molecular function, biological process, and cellular distribution of proteins unequivocally expressed in MVs and Exos fractions, we imported data produced by the MS identification in the Protein Analysis Through Evolutionary Relationship (PANTHER) and Gene Ontology (GO) databases. Identified proteins were further analysed using the software STRING (http://string-db.org/ (accessed on 2 February 2021)), chosen as the source for protein–protein interactions, to statistically determine the functions and pathways most strongly associated with the protein list. This program builds protein networks based on known direct and indirect interactions described in literature. A confidence level of 95% was considered the cut-off for the analysis.

### 2.9. Data Analysis

All experiments were carried out in at least two independent biological replicates and processed for statistical significance as indicated. Whenever applicable, numerical values are reported as mean ± S.D. Differences were considered statistically significant at *p* < 0.05 (*t* Student, one way).

## 3. Results

### 3.1. Isolation of Two EV Subtypes by Sequential Centrifugal Ultrafiltration (SCUF) Technique and Their Characterization by Transmission Electron Microscopy (TEM) and Western Blot Analysis

We isolated two subtypes of EVs by applying the SCUF technique (as reported in the Methods section) to CM removed from cultured GSCs derived from primary GBM of two patients. Of note, this CM did not contain serum, since GSCs normally grow without this supplement. Thus, plasma contamination was avoided. Additionally, the first centrifugation adopted in our experimental protocol removed possible cell debris. By SCUF, we separated two main fractions, which were called Fn1 and Fn5 (Figure 1) and were processed by electron microscopy procedures to obtain their ultrastructural characterization.

**Figure 3:**
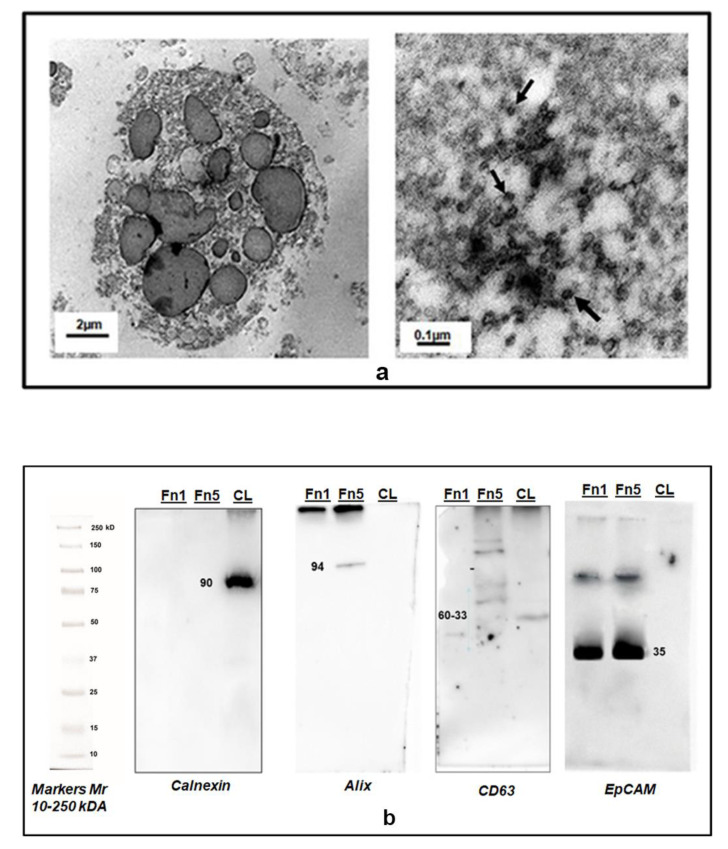
Characterization of EVs isolated from the CM of GSCs by transmission electron microscopy (TEM) and Western blot analysis. (**a**) Representative TEM images of two different types of EVs isolated from the total secretome of human GSCs. Left panel: MVs (Fn1 fraction) in the size range of 100–1000 nm and above. Right panel: Exo-like vesicles (some of which are indicated by black arrows, Fn5 fraction) in the size range of 30–100 nm. (**b**) Western Blot analysis of 30 µg of proteins from isolated EVs confirmed Fn5purity for the presence of canonical exosome proteins like Alix and CD63 and the absence of Calnexin, detectable only in the whole cell lysate (CL). Fn1 strongly reacted with anti-Abs to EpCAM, but no response was visible as for Alix or CD63. Mr = molecular range of the weight of proteins, expressed as kiloDaltons (kDa), revealed by the appropriate antibodies (see Methods Section).

As shown in Figure 3a, both types of vesicles were round-shaped. Vesicles in Fn1 were present in a small number and appeared as large particles up to 1000 nm, with morphological features that could also be compatible with those referred to oncosomes, as reported by others’ studies [4,33]. Instead, vesicles in Fn5 fraction were more homogeneous, surrounded by an amorphous matrix, and typically cup-shaped with internal diameter <100 nm. The same Fn1 and Fn5 fractions were also characterized by Western blot analysis (Figure 3b); the lanes related to Fn5 reacted to antibodies against Alix, CD63, and epithelial cell adhesion molecule (EPCAM) and accepted Exo markers, whereas lanes for Fn1 showed a positive reaction only towards EPCAM.

### 3.2. Proteomic Analysis of Exo and MV Content

2DE analysis was carried out on total cell extracts (as indicated in the Figure 2). The total protein yield of the EVs from the two GSCs was approximately 1.7 ± 0.012 and 0.6 ± 0.004 mg (n. of tested samples for each GSC type = 3) for MV and Exo fractions, respectively.

**Figure 4:**
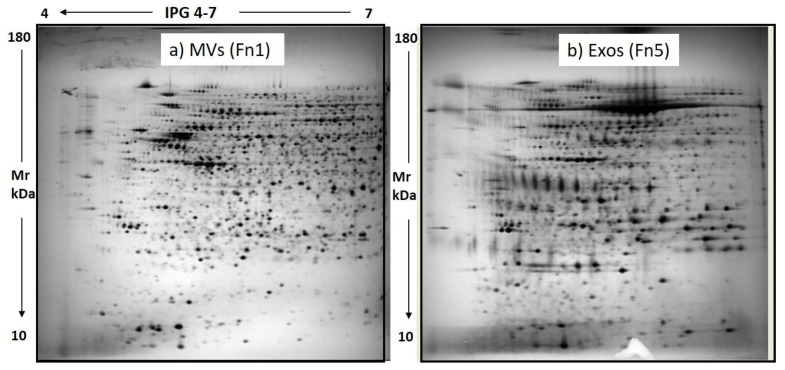
Representative 2D maps of GSC EV subpopulations, MVs (**a**) and Exos (**b**). 150 μg from SCUF-derived MVs and Exos were resolved by 2D PAGE on 4–7 IPG strip (24 cm) on 12% homogeneous gel.

For each extracted sample, 150 µg of total proteins were loaded on 12% homogeneous gel at a 4–7 pH gradient. By this method, the 2D electrophoretic run resolved 1933 ± 106 and 1625 ± 98 protein spots for the Fn1 and Fn5 samples, respectively, distributed across 4–7 pH range. 2D maps representative of the Fn1 (MVs) and Fn5 (Exos) fractions are shown in Figure 4.

Of note, the image analysis of these gels using extracts from the vesicular fractions of GSCs isolated from two human GBMs revealed a similarity in the protein pattern greater than 85%. The high reproducibility of the 2D maps was confirmed by the number of resolved protein spots and matching % between gels from each condition (Fn1 and Fn5).

**Figure 5:**
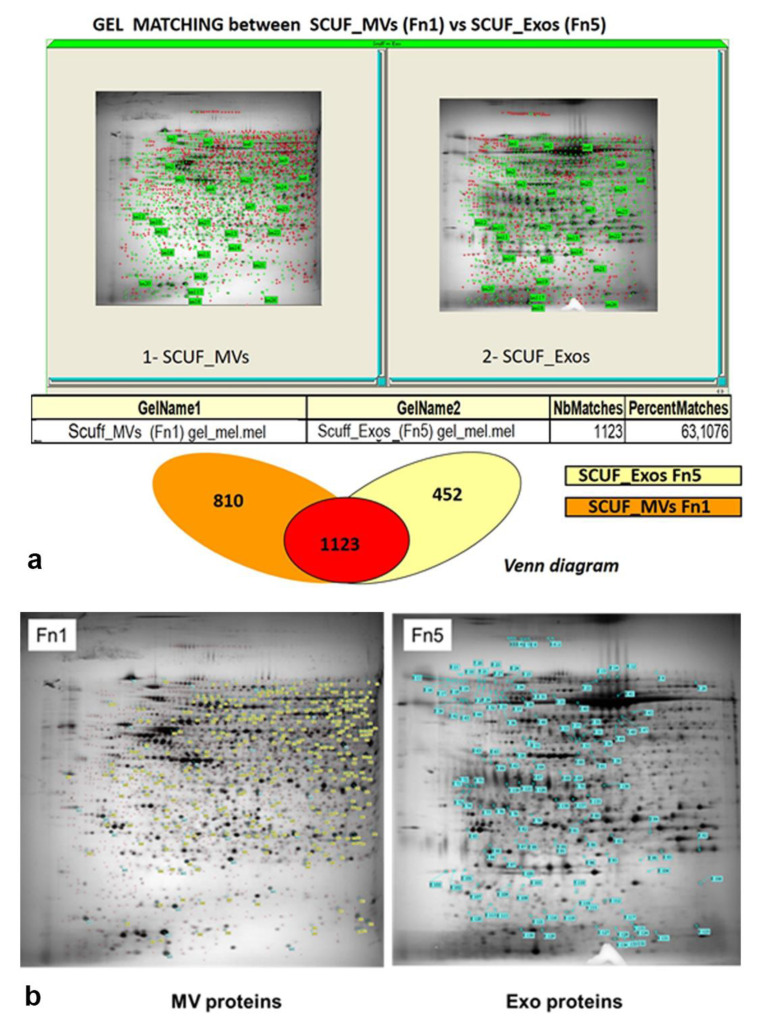
Identification of the major protein spots on gels run using extracts from MV and Exo fractions of GSCs and comparison between spots from the two fractions. (**a**) Gel matching between SCUF-MVs and SCUF-Exos (statistical analysis of gel similarity) and Venn diagram showing common and unique proteins detected in at least three technical replicas in the Fn1 and Fn5 vesicle-enriched fractions; (**b**) 2DE profiles of GSC EV subpopulations, MVs (Fn1) and Exos (Fn5). All proteins exclusively expressed in each fraction, 245 for MVs and 137 for Exos, are marked with yellow and light blue labels, respectively.

The % matching, a statistical analysis of gel similarity, showed a significant overlap between MV and Exo proteomes (Figure 5a), since about 63% of the proteins, equal to 1123 ± 52 spots, were common to both fractions. This was probably due to the presence of proteins constitutively expressed by the examined GSCs that characterized their total secretome, even though with some differences in their expression level. The comparative analysis between master gels also highlighted an average ± S.D. of 810 ± 64 exclusive spots for the Fn1 sample, while the specific protein spots for the Exo fraction (Fn5) were 452 ± 26. These differences characterizing the two fractions were likely due to the different origin of EVs from cells and/or induction of their secretion.

Among protein spots highly expressed in each EV type, we selected only those with a statistically significant intensity value (*p* < 0.05) and expression level ≥2 for the subsequent identification in MS. By this method, 245 ± 20 and 137 ± 11 protein spots were picked from MVs and Exo gels, respectively (Figure 5b).

Subsequently, differently expressed (*p* < 0.001) protein spots were submitted to in-gel tryptic digestion and identification by MALDI-TOF-TOF mass spectrometry (MS). Only proteins 21 and 9, exclusively present in the Fn1 or Fn5 fractions with a significant dysregulation level value, were identified by MS; these results are listed in the Table 1a,b. Proteins identified by MS as dihydropyrimidinase in MVs and Exos fractions as S114 and as E48 are indicative of two different isoforms that have different pI. Additionally, proteins labeled as S148 and S184 represent two isoforms belonging to vimentin family, that show the same MW, but different pI.

### 3.3. Identification of Some Proteins Exclusively Present in MV Fraction

A great number of the top proteins identified in the MV fraction (Table 1a) derive from cell organelles such as nuclei and mitochondria or structures like cytoskeleton. Thus, lamin B1(LMNB1) and prelamin-A/C (LMNA) belong to the family of Lamin proteins that are located in the nuclear membrane with the general function of stabilizing the binding of proteins and chromatin [34]. Additionally, X-ray repair cross-complementing protein 6 (XRCC6) is another nuclear protein like heterogeneous nuclear ribonucleoprotein H1 (HNRH1), a member of the family of heterogeneous nuclear ribonucleoproteins (hnRNPs) that contribute to multiple aspects of nucleic acid metabolism, thus playing key roles on development/differentiation of mammalian cells [35]. Again, eukaryotic translation elongation factor 1 delta (EEF1D) is a subunit of the elongation factor 1 (eEF1) complex, which mediates the elongation process in the eukaryotic protein synthesis [36]. In relation to mitochondria, we detected cytochrome b-c1 complex subunit 1 (QCRC1), which has a fundamental role in aerobic cell metabolism; Mitochondrial Translation Elongation Factors Tu (EFTU), one of the most abundant proteins of mitochondria participating in polypeptide biosynthesis of these organelles [37], and glucose-regulated protein 75 (GRP75), also known as Mortalin, involved in intracellular transport, cell proliferation, stress reaction, and cytoskeleton stabilization [38]. As for cytoskeleton, top MV proteins were vimentin (VIME), dihydropyriminidase-related protein 2 (DPYL2), and moesin. VIME is one of the most widely expressed and highly conserved proteins of the type III intermediate filament (IF) protein family that contributes to maintain cell integrity and resistance against stress; moesin is a member of the Ezrin–radixin–moesin (ERM) protein family that connects actin to the plasma membrane, thus regulating structure/function of specific domains of the cell, whereas DPYL2 promotes microtubule assembly, playing a major role in neuronal development and polarity, as well as in axon growth and guidance and cell migration.

Other top MV proteins showed multiple cell locations, but could be grouped based on their function. Indeed, many of them belong to the family of chaperonins. Besides the aforementioned GRP75, we detected Heat Shock cognate 71KDa Protein (HSP7C), which is one of the major constituents of the Epidermal Growth Factor Receptor (EGFR) complex [39] and, like heat shock 70 kDa protein 1A (HSP71A), is also an essential regulator of cellular protein quality control, therefore being one of the most important players in the endoplasmic reticulum processing [40]. As well, peptidyl-prolyl-cis-trans isomerase FKBP4 (also known as FKBP52) and peptidyl-prolyl cis-trans isomerase (FKBP5) are immunophilin proteins with co-chaperone activities. Likewise, T-complex protein 1 subunit zeta (TCPZ) is known as chaperonin-containing T-complex polypeptide 1 (CCT) or TCP1 ring complex. We also determined the presence of calmodulin lysine methyl transferase (CaMKMT), which is a highly conserved protein whose expression in humans is required for muscle growth and brain function [41].

Finally, we detected the protein sequence of enzymes involved in cell metabolism like aldehyde dehydrogenase 3A1 (AL3A1), an important member of the aldehyde dehydrogenase superfamily comprising enzymes able to oxidize endogenous/exogenous aldehydes to the corresponding carboxylic acids [42]; aldo-keto reductase family 1 member 1 (ALDR1 also known as AKR1B1), belonging to the aldose keto reductase (AKR) superfamily [43]; and glutathione synthetase (GSS), a well-known protein involved in the pathway leading to synthesis of glutathione from L-cysteine and L-glutamate.

Noteworthily, as briefly described, all aforementioned proteins play important roles in normal cell biological functions. However, they have mostly been detected and/or abnormally expressed in different cancers [44,45,46,47,48,49]. Thus, their inappropriate presence or function may contribute to the cancerogenic process or to resistance to chemotherapy with a worse clinical outcome [50,51].

### 3.4. Identification of Some Proteins Exclusively Present in the Exo Fraction

Looking at the specific proteins more expressed in Exos (Table 1b), apart from DPYL2, they were different from those identified in MV proteome as top proteins. Some of them are mostly secreted from cells, such as clusterin (CLUS), procollagen III (CO3A1), and complement C1s subcomponent (C1S). However, we also detected proteins from cytoskeleton (T-complex protein 1 subunit theta, TCPQ, and DPYL2), cell nucleus (mirror-image polydactyly gene 1 protein, MIPO1, and DNA-directed RNA polymerase II, subunit RPB11-a, RPB11), mitochondria (ATP synthase subunit beta, ATPB), or cytosol (protein S100A14, S10AE).

Among the last ones, MIPO1 has been associated to craniofacial development, whereas the related gene aberration is coupled to congenital anomalies [52]. In contrast, the DNA-directed RNA polymerase II subunit RPB11-a is a part of the core element of the RNA polymerase II that synthesizes mRNA precursors and many functional non-coding RNAs. Again, the cytosol protein S100-A14, which modulates P53/TP53 protein levels, contributes to regulating cell survival and apoptosis, whereas CCT theta is a eukaryotic cytosolic protein assisting in the folding of proteins as a chaperonin. Finally, ATP synthase subunit beta together with the subunit alpha forms one of the two structural domains of ATPase, which is linked to mitochondrial membrane and produces ATP from ADP in the presence of a proton gradient across the membrane generated by electron transport complexes of the respiratory chain [53]. As for MV top proteins, those selected in Exos have also mostly been detected in different tumors, likely playing a role in cancer progress [54,55,56,57,58,59,60,61,62,63].

### 3.5. Functional and Biological Analysis of the Proteome of Isolated Exo and MV Fractions

**Figure 6:**
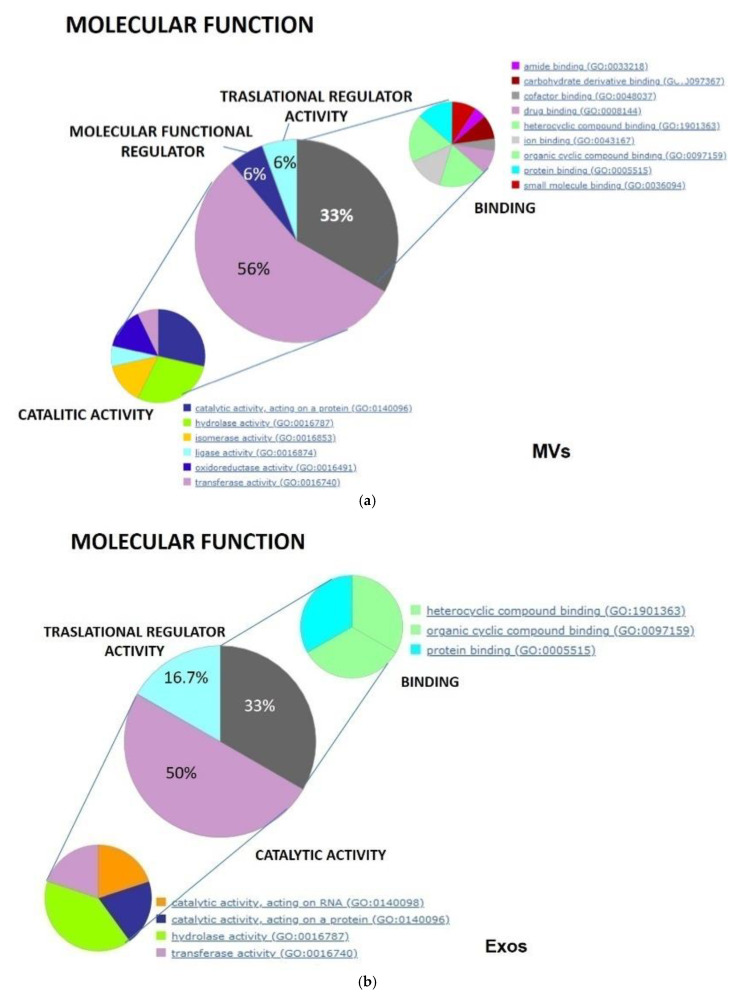

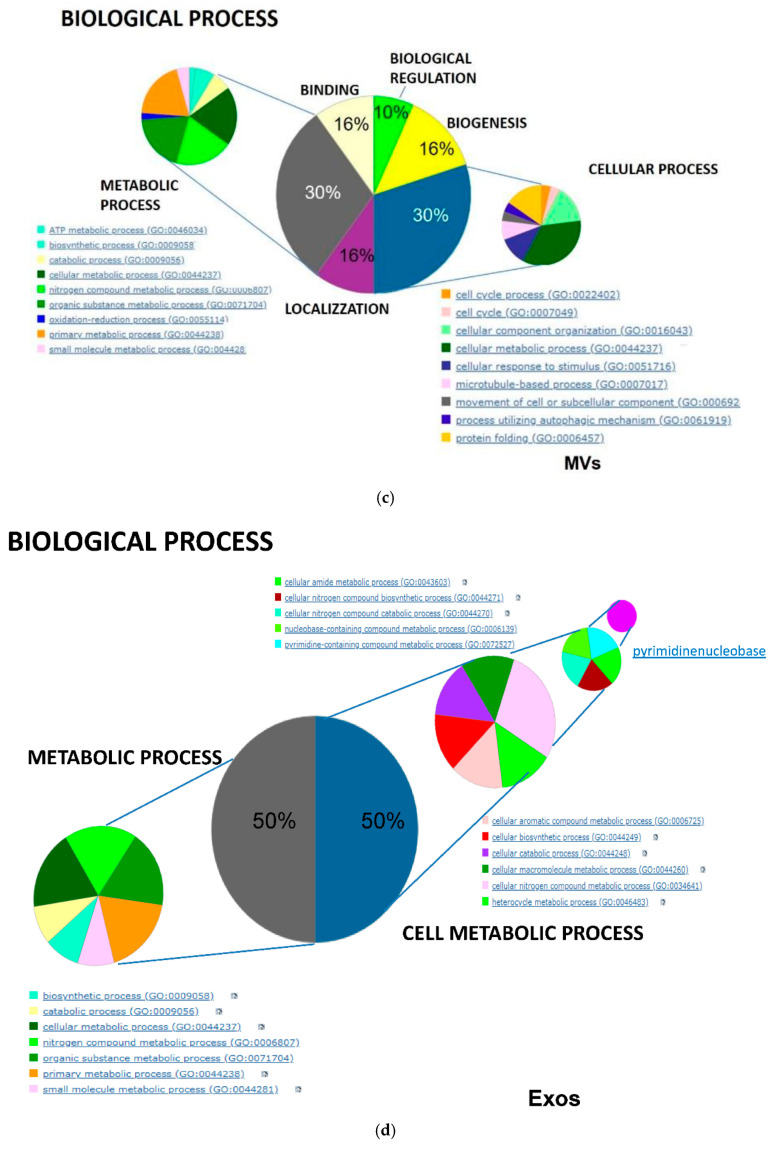

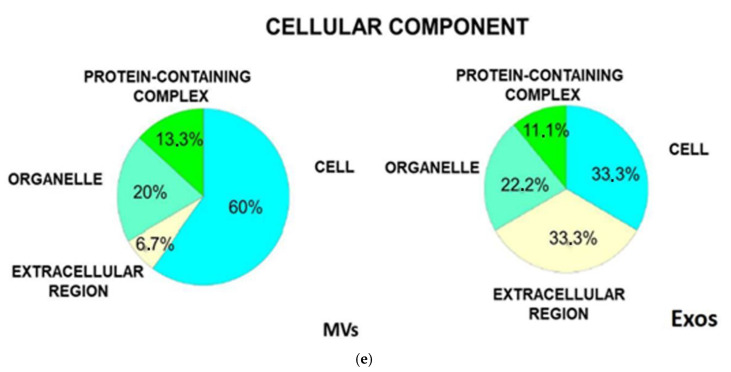
Analysis by Gene Ontology of the specific proteins for each type of EVs isolated from the CM of GSCs. This analysis allowed us to distinguish the percentage of those proteins depending on different molecular function (**a**,**b**), involvement in diverse biological processes (**c**,**d**), and cellular localization (**e**).

Data produced by MS analysis and reported in the Table 1 were also analyzed by importing them in the GO and PANTHER database. This analysis gave a more general view, confirming the molecular function and the biological processes in which the top selected proteins are involved, specifically expressed in the GSC-derived MVs and Exos. Indeed, as highlighted by the pie charts (Figure 6a,b), MV and Exo protein distribution was characterized by prevalent catalytic (about 50%) and binding (33%) activities. Additionally, in MV, a similar small percentage of proteins were deputed to molecular or translational regulator activity (6%), whereas in Exos, a significant percentage of proteins (about 17%) were grouped as provided with only translational regulator activity. Looking at the specific activity of the identified proteins, in MVs (Figure 6a) there was a large variety of proteins with different activities compatible with MV derivation from cell membranes such as drug or ion binding, chaperone, or transporters. In Exos, the most abundant proteins showed hydrolase and also nucleic acid/protein catalytic activity whereas others, expressed to a lesser extent, included cytoskeletal proteins, enzyme modulators, chaperones, calcium binding proteins, transferases, and transporters.

As well, the comparison of the % distribution of the vesicular proteins among biological processes (Figure 6c,d) revealed a different involvement of MV or Exo proteins compatible with their derivation from diverse cellular compartment. This analysis confirmed that MV proteins exhibited a more heterogeneous distribution, with most proteins deputed to metabolic or cellular processes, whereas a smaller percentage of them was involved in localization or response to stimuli or involved in cell regulation processes. In contrast, Exo proteins were equally distributed between metabolism and cellular processes, with a lower percentage of these latter with a role in developmental processes.

Finally, the analysis of protein distribution relating to cellular components (Figure 6e) showed a greater percentage of proteins from cells and a lower rate of those from the extracellular region in MVs in comparison to Exos, whereas the distribution of proteins derived from organelles (about 20%) or belonging to multiprotein complexes (11–13%) was similar in both vesicle types.

## 4. Discussion

In hundreds of recently published scientific articles, GBM is defined as the most aggressive and malignant primary human brain tumor with a very poor diagnosis and availability of effective therapeutic treatments. Hence, the urgent need to find easily detectable biomarkers that can make GBM more precociously identifiable and/or represent new possible druggable targets. In this context, the study of EVs is raising a great interest in different types of cancer including GBM, making available a large amount of information and promising results, especially in the field of diagnosis [1].

Relevant to the above issue, our study presents several points of interest. First, our investigation was carried out on cells directly deriving from primary human tumors. They have been characterized as GSCs, which play a key role in tumor mass growth and invasion [64]. Although conventional GBM cell lines (mainly cultured under serum-based media conditions) are widely used to investigate also EV content, mutations may occur during long-term culture in serum-containing media that could bias the results [11]. In contrast, GSCs used in our study derive from freshly resected tumor specimens and are cultured in serum-free medium, under conditions optimized for the growth of neural stem cells. In addition to the fact that GSCs more closely mirror the genotype/phenotype of primary tumors than serum-cultured cell lines, the lack of serum in the growth medium represented a great advantage to our purposes, excluding possible external contamination to the pattern of proteins isolated from GSC EVs. It is also important to recall that the molecular profile of the GSCs used here has previously been reported [21] and can be related to the patients’ outcome and response to TMZ in malignant gliomas [65]. Nevertheless, the pattern of the EV proteome for the two cell types was very similar. Of course, we are aware that findings from our study should need to be confirmed in EVs from a wider number of human GBM-derived GSCs.

Another important aspect of our study is that we aimed at isolating and studying two EV types from the CM of GSCs. Most articles published so far are mainly addressed to elucidate the proteome and, possibly, the biological and functional role of Exos. However, different types of EVs are released from virtually all cells, even though their list can be reduced to two main subtypes that are micro- (MVs, 100–1000 nm) and nano-sized (Exos, 30–150 nm) vesicles, both representing a highly sophisticated system to exchange biological information with close or distal cells [66]. Therefore, it seemed of interest to isolate and characterize in parallel the two different EVs from our GSCs. The method, partially modified by the procedure of Xu et al. [28], showed the following key features: effectiveness, reproducibility, and suitability for different starting materials. Therefore, we do believe that this protocol, among many others commonly used to isolate EVs, may have a good application in this research field.

TEM analysis, which currently is the gold standard technique for the classification of EVs into size and shape different categories, confirmed the membrane integrity and measured the size of the particles isolated by SCUF, whereas Western blotting documented the presence of selected markers for MVs and Exos, as previously found in EVs from GBM [15,19] or other cancers [28]. The same finding also excludes that our EVs may be apoptotic bodies.

Once MVs and Exos were obtained and identified, further screening was performed to characterize and compare the protein profile of these EVs. The 2D electrophoretic analysis detected a great number of specific proteins for MVs that was roughly double that identified in Exos, although more than one thousand proteins were common to both EV types. However, looking at the intensity of expression and to the related statistical significance, those numbers were drastically reduced to about a quarter of the initial amount in each EV fraction. By this method, our data appear to be similar to those obtained from other research groups, who mostly used GBM cell lines and/or analyzed the proteome of Exos [15,67,68].

As expected from the Venn diagram, a discrete amount of specific proteins with different biological/functional profiles were present in the two EV types. Among these, MVs were characterized by a major presence of cell proteins, which were also found in Exos but in balance with proteins from the extracellular region (secreted). This finding would suggest that MVs could be regarded also as “oncosomes”, even though their measured size was lower than that indicated in literature for these vesicles (1–10 µm) [4,33]. However, the diversity of the identified proteins, apart from the origin of MVs or Exos from different cell compartments [4], could be ascribable to possible different functions exerted by them, once released in vivo.

In relation to this aspect, in MVs we found proteins previously reported in GBM EVs [67,68] and/or able to influence GBM aggressiveness [69,70,71]. Among them, there were a large number of chaperones belonging to the HSP family, which regulate the appropriate protein folding in normal cells, while showing altered expression/activity during stress, a condition characterizing also growth/expansion of tumors [72], including GBM [73]. In particular, we revealed an abundance of proteins related to the HSP70 family, which also includes GPR75, usually localized in mitochondria. Moreover, we detected FKBP4, the upregulated expression of which was also found in GBM [56]. Additionally, the presence of Lamin A/C isoforms together with HSP was found in GBM cell lines U87, which may represent an index of disease progression [74,75].

Interestingly, drawing a network among these proteins (Figure 6), it emerges that most of them are intimately connected. However, for some chaperone proteins identified in our MVs such as FKBP5, TCPZ (both present in the network of the Figure 6), or also CaMKMT, it would be important to confirm their presence in MVs from a wider GSC number and to evaluate their role via EVs in GBM growth or progression.

Our data also highlighted in MVs a large presence of enzymes linked to cell metabolism that, so far, have been detected in brain tumor Exos, representing about 25% of the total proteins therein detected [76]. In particular, it is to underline the presence of GSHB, an enzyme deputed to glutathione synthesis from L-cysteine and L-glutamate. Interestingly, glutathione levels in GBM cell lines have previously been related to drug resistance [77]. Additionally, it is important the detection of *UQCR1*, which can interconnect with other enzymes deriving from mitochondria (Figure 6). Accordingly, previous studies demonstrated that large EVs from tumors transport proteins targeted to mitochondrial metabolic processes [33]. Thus, these data suggest that MVs also have intrinsic metabolic capacity that could be of support to the growth of neighboring cancer cells. More in general, since all these proteins are usually regarded as intracellularly localized, the fact that they can be transported by MVs might change their role to biomarkers or, if acquired by silent tumor cells, they could reinforce their aggressiveness.

**Figure 7:**
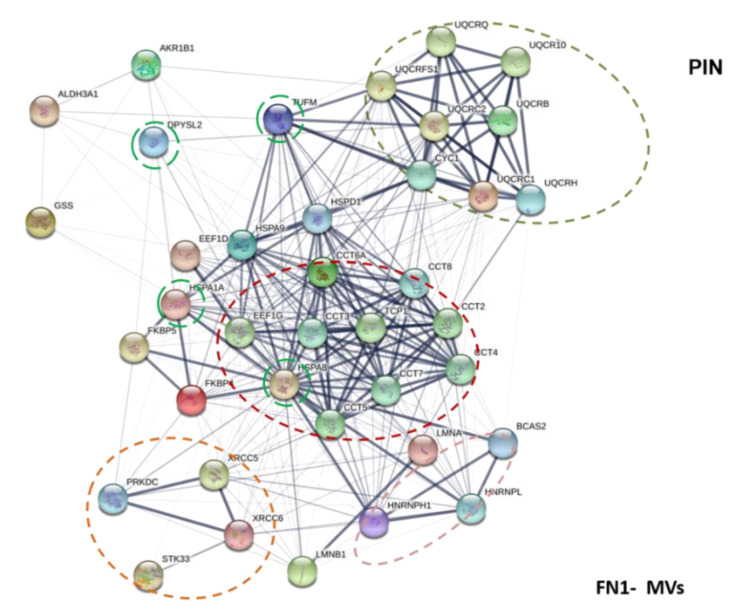
MV Protein Interaction Network. Functional links of proteins exclusively expressed in MVs fraction using STRING were constructed (http://string-db.org (accessed on 2 February 2021)). Proteins shown as spheres and labeled with gene name represent the nodes, whereas nodes that are associated to each other are linked by edges that represent their interaction. Thicker lines are related to a stronger association. TUFM, DPYSL2, HSPA1A, and HSPA8 are hub proteins (a node with a number of about four and/or five edges). Main clusters of predicted molecular pathways are indicated with colored circle.

Of note (Figure 7) is the possibility that the cytosolic enzymes (found in MVs, Table 1a) are interconnected among themselves and also with DPYSL2, which is, together with VIME (also identified in MVs), a cytosolic protein not expressed in normal brain tissue, whereas its levels as well as those of VIME are significantly high in GBM [69]. VIME, in turn, can connect cytosolic to mitochondrial enzymes via EFTU (also called TUFM) that seems to play a central role behaving as a hub protein, also able to interconnect with other chaperone proteins found in MVs (Table 1a). EFTU/TUFM normally performs various functions including cell morphology and transformation, organization of mitotic apparatus, developmental regulation, cytokine response, and increase of autophagy [78,79]. Growing evidence has indicated that dysregulation of TUFM is involved in the oncogenic process in various tumors [80], whereas there is very recent evidence in GBM [69].

Finally, for the first time, to our knowledge at least, our study detected in Exos (Table 1b) a discrete number of proteins as specifically expressed in particles deriving from human GBM. If confirmed by further studies, they might represent distinctive features to be used as potential biomarkers/druggable targets. This may be the case of CO3A1or RPB11, which have so far been identified as involved in the progression of other cancer types [44,59,60], whereas studies on the role of complement C1s or MIPOL1in cancer are still poor [61,62].

**Figure 8:**
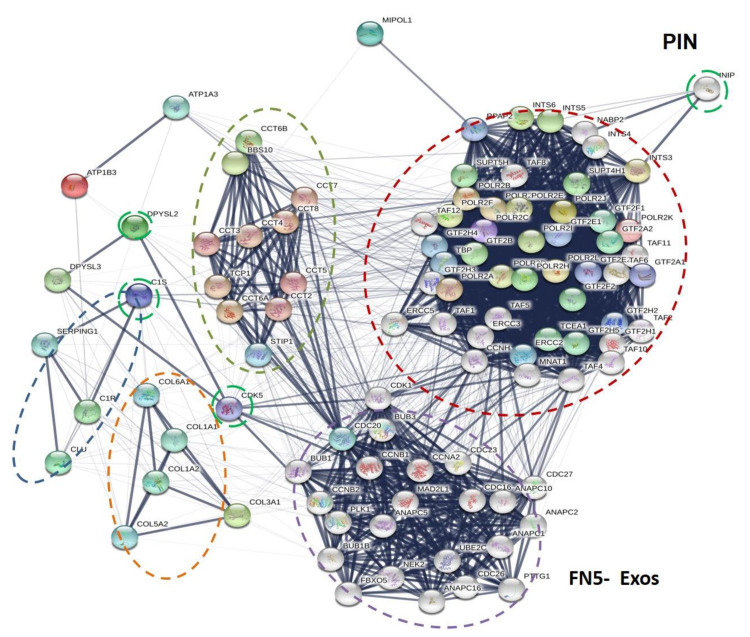
Exo Protein Interaction Network. Association of proteins exclusively expressed in Exo fraction using STRING were constructed (http://string-db.org (accessed on 2 February 2021)). Proteins shown as spheres and labeled with gene name represent the nodes, whereas nodes that are associated to each other are linked by an edge, which represent their interaction. Thicker lines are related to a stronger association. DPYSL2, CCT8, C1S, CDK5, and INIP are hub proteins (a node with a number of about four and/or five edges). Main clusters of predicted molecular pathways are indicated with colored circle.

Looking at the possible network of these proteins with other ones (Figure 8), it is evident the relationship of the first four proteins with others belonging to the same class.

However, the chaperone family, including TCPQ (related gene *TCP1*), is, in turn, intensely related to a dense network of proteins involved in promoting or controlling cell proliferation. Of interest, DPYSL2 is also present among Exo proteins and, once again, it might play a central role, being connected with cyclin-dependent kinases (CDKs), a family of protein kinases first discovered for their role in regulating the cell cycle and considered as potential target for anti-cancer medication also in GBM [81]. In contrast, MIPOL1 is uniquely linked to another dense network of proteins translating the nuclear message.

Altogether, these data suggest that Exos proteins, if acquired from quiescent tumor cells, could promote their proliferation and consequently GBM growth or recurrence. Such a possibility is reinforced by examining the activity of other proteins detected in Exos. While the presence of moesin, a cytosolic protein associated with unfavorable patients’ survival in various cancers, has already been reported in GBM [70], CLUS expression has been identified in breast cancer cells as an extracellular protein that, interacting with extracellular HSP90 protein, promotes tumor metastasis [82], or the overexpression of which induces chemotherapy resistance in human gastric cancer cells [83]. The same is valid for the S100-A14 protein, which has so far been described as a breast cancer biomarker [84]. However, it is to underline that, in gliomas, this protein contributes in vivo to recruit myeloid cells including microglia and monocytes, thus providing a tumor supporting environment. This finding would confirm the role of EVs in tumor immune escape [85]. Additionally, it is to clarify the role of the ATP synthase subunit beta present in our GBM EVs, since when it is ectopically expressed on cell membranes of different cancers seems to support the tumor growth and metastasis [63,86].

## 5. Conclusions

In conclusion, our study has identified two main types of EVs from the CM of GSCs deriving from human primary GBM, which differed in terms of size and transported signals. This last aspect is of great significance, since it may lead to the modulation of a range of cellular functions. Indeed, our data suggest that MV proteins, mainly acting as chaperones or metabolic enzymes, would offer a protective system and a metabolic support to tumor cells against stressful inputs from environment occurring during tumor expansion, whereas Exo proteins could supply cells with molecules important for cell–matrix adhesion (procollagen III), cell migration and aggressiveness (moesin, S100-A14 protein), or resistance to anticancer drugs (DNA-directed RNA polymerase II, subunit RPB11-a), possibly cooperating with MV proteins with similar activity (lamin B1 and vimentin).

Based on literature, it is possible to affirm that overexpression of numerous proteins isolated from GBM-derived MVs or Exos had already been found in cells from peripheral cancers; however, we deem that their expression needs to be confirmed in a greater number of GSCs from different human GBMs and, as a consequence, of GSC-derived EVs to validate their role as pro-tumoral agents or useful prognostic or druggable biomarkers.

## Figures and Tables

**Table 1:** List of GSC EV proteins identified by MS analysis.

**(a) Top Proteins Identified in the MV Fraction from the CM of GSCs**								
**SPOT** **ID**	**Abbr.** **Name**	**AC a Swiss/** **NCBI**	**Protein Description**	**Score b**	**Peptide Matched**	**SC c %**	**Theoretical** **(pI/Mr)**	***p*-Value**
S53	MOES	P26038	Moesin	194	53	65	6.08–67.89	0.0001
S77	GRP75	P38646	Stress-70 protein, mitochondrial	111	49	55	5.87–73.92	0.0013
S81	XRCC6	P12956	X-ray repair cross-complementing protein 6	42	17	31	6.23–70.08	0.0021
S84	HSP7C	P11142	Heat shock cognate 71	50	33	40	5.37–71.08	0.0011
S92	HS71A	P0DMV8	Heat shock 70 kDa protein 1A	66	21	36	5.48–70.29	0.0034
S100	LMNB1	P20700	Lamin-B1	76	26	36	5.11–66.65	0.0016
S114	DPYL2	Q16555	Dihydropyrimidinase-relatedprotein 2	52	18	29	5.95–62.71	0.0003
S148	VIME	P08670	Vimentin	129	55	76	5.06–53.67	0.0024
S115	LMNA	P02545	Prelamin-A/C	81	29	41	6.57–74.38	0.0008
S132	TCPZ	P40227	T-complex protein 1 subunit zeta	34	27	44	6.24–61.59	0.0010
S153	FKBP4	Q02790	Peptidyl-prolyn cis-trans isomerase	50	29	48	5.35–52.05	0.0073
S160	AL3A1	P30838	Aldehyde dehydrogenase, dimeric NADP-preferring	41	11	18	6.11–50.76	0.0001
S168	HNRH1	P31943	Heterogeneous nuclear ribonucleo protein H1	119	28	54	5.89–49.48	0.0022
S169	FKBP5	Q13451	Peptidyl-prolyn cis-trans isomerase	36	18	38	5.70–51.69	0.0016
S179	GSHB	P48637	Glutathionesynthetase	40	36	60	5.67–52.52	0.0002
S191	EFTU	P49411	Elongation factor Tu, mitochondrial	119	31	68	7.26–49.85	0.0040
S181	QCR1	P31930	Cytochrome b-c1 complex subunit 1, mitochondrial	165	34	61	5.94–53.29	0.0017
S184	VIME	P08670	Vimentin	80	36	59	5.06–53.67	0.0037
S210	CMKMT	Q7Z624	Calmodulin-lysine N-methyltransferase	26	7	17	6.37–36.78	0.0056
S254	ALDR	Q9UBJ2	Aldo-keto reductase family 1 member B1	118	21	52	6.51–36.23	0.0001
S259	EF1D	P29692	Elongationfactor 1-delta	56	21	60	4.90–31.27	0.0008
**(b) Top Proteins Identified in the Exo Fraction from the CM of GSCs**								
**SPOT** **ID**	**Abbr.** **Name**	**AC ^a^ Swiss/** **NCBI**	**Protein Description**	**Score ^b^**	**Peptide Matched**	**SC ^c^ %**	**Theoretical** **(pI/Mr)**	***p*** **-Value**
E23	C1S	P09871	Complement C1s subcomponent	63	27	42	4.87–99.06	0.0021
E46	TCPQ	P50990	T-complex protein 1 subunit theta	58	24	43	5.26–61.26	0.0015
E48	DPLY2	Q16555	Dihydropyrimidinase-related protein 2	115	32	66	5.95–62.71	0.0008
E51	MIPO1	Q8TD10	Mirror-image polydactyly gene 1 protein	43	22	49	5.55–51.84	0.0019
E54	ATPB	P06576	ATP synthase subunit beta, mitochondrial	66	17	42	5.26–56.52	0.0005
E120	S10AE	Q9HCY8	Protein S100-A14	30	5	50	5.16–11.82	0.0018
E130	RPB11	P52435	DNA-directed RNA polymerase II subunit RPB11-a	47	6	55	5.63–13.34	0.0043
E137	CO3A1	P02461	Carboxy-propeptide of alpha 1 (III) procollagen	70	22	46	5.89–27.90	0.0005
E139	CLUS	P10909	Clusterin	75	12	38	5.89–53.03	0.0001

All the identified proteins relate to *HOMO SAPIENS.19453.* ^a^ AC is the accession number. ^b^ Score is −10*Log(P), where P is the probability that the observed match is a random event; it is based on Swiss-Prot/NCBI database using the MASCOT search engine. ^c^ Sequence coverage means the ratio of portion sequence covered by matched peptide to the full length of the protein sequence.

## Data Availability

Data are available upon request by qualified researchers to the Corresponding Author.
